# A Plea for the More Extended Use of Spectacles as a Therapeutic Measure

**Published:** 1890-12

**Authors:** Frank Trester Smith

**Affiliations:** Chattanooga, Tenn.


### A PLEA FOR THE MORE EXTENDED USE OF SPECTACLES AS A THERAPEUTIC MEASURE.*

FRANK TRESTER SMITH, A. M., M. D., CHATTANOOGA, TENN.

One could hardly hope in a brief paper to exhaust a subject so extensive, nor could one expect in such a learned body to instruct, for I have nothing specially new to present; but if I can show the subject in a new light, or if I can get you to consider the matter, the object of this paper will be accomplished. In hospital practice our staff regarded it as a duty due the patient to examine every organ in all obscure cases; the ears, the eyes, the nose, the respiratory and digestive tracts, the genital organs, the muscular system, etc., were all interrogated systematically

*Read before the Mississippi, Valley Medical Association.

whenever we were puzzled for a diagnosis. That many physicians are content with an incomplete diagnosis or none at all, in many cases that would be cleared up by the above procedure goes without saying. It is not unknown that Bright's disease has been diagnosed by the ophthalmoscope. The cause of a persistent cough has been found in the ear; nasal hypertrophies have been discovered to be the cause of many reflex disturbances.

These are truisms which need hardly be repeated.

During the past few years much has been written on the subject of reflex ocular disturbances, and owing to the extravagant claims of some which have not been sustained by more extended observation, I fear the matter has fallen into disrepute, or that it is regarded rather as a theoretical than as a practical matter. We are liable to run to extremes, and while the subject of reflex ocular troubles may not be the cause of all disturbances that enthusiasts claim that they are, they are of much more consequence than is appreciated by many general practitioners. This paper will deal only with those disturbances which may be relieved with the use of glasses, more particularly with the indications of the use for spectacles as a therapeutic measure.

Spectacles may be divided into the following classes: protective prisms and refractives, the latter including spherical and cylindrical lenses. These are variously combined.

The most frequent indications for which spectacles are used is presbyopia, so called "old sight." For this the patient generally prescribes himself. That is he goes to a jewelry store and picks out a pair of glasses that seem to fit. In uncomplicated cases no great harm can come, but when these simple glasses do not give ease the patient should be urged to have the eyes examined by an expert. Too often they give up the use of the eyes at night, sometimes by the advice of a physician. They are thus needlessly deprived of much valuable time, for the majority of these cases can be made to read with comfort by fitting the proper lenses. These cases are often found to be astigmatic, for which a cylindrical glass is needed; then with a spherical to correct the presbyopia they read with comfort. The proper combination is not always found at the first attempt.

Probably the most frequent indication for which the oculist is consulted is diminished vision. These cases naturally drift to the specialist. But it is a strange fact that there are some who have much less than normal vision, but who are not aware of the fact. They suppose that every one sees as they do. This is well illustrated in a case that came under my observation a few years ago. A young man eighteen years of age happened to try on a pair of concave spectacles, and was much surprised to find with what clearness and distinction he could see distant objects. The glasses were a perfect revelation to him. He lost no time in securing a pair. He had supposed that he saw as well as any one. More especially where one eye is defective is it apt to be overlooked, unless accidentally discovered.

In that large group of cases which is classified under the head of asthenopia indication for an examination of the refraction is so apparent that one would naturally imagine that glasses would be the first thought of the physician, but not so. In the case of children the hope is sometimes held out that they will outgrow the difficulty; in adults the fear is excited that if they begin the use of glasses they will have to wear them always. In headache spectacles should always be thought of. An ocular headache is generally brought on or increased by use of the eyes, but this is not necessarily the case; sometimes there is no indication that the eyes are at fault. Again in some cases the proper lenses will not cure at once the headache. They must be worn for some time This use of glasses is one of the triumphs of the medical art, and yet there are many who are not availing themselves of it. I recall just now one family in particular where a mother and daughter are frequently prostrated by the most severe headache, but because some might think they were wearing glasses for style they refuse the only means that promises a cure.

There are some reflex disturbances other than headache which would be benefited by glasses; as a rule a careful history will bring out the fact there have been eye symptoms.

In obscure cases the refraction of the eye should be tested before the final diagnosis is made.

I have thus called your attention to some of the main indications for the use of spectacles. Having determined that an examination is advisable, to whom will you entrust this work; sometimes it is sent to the jeweler or the optician. The distinction between the oculist and optician is not always observed. the latter may know much of optics and glasses and the needs of the eye, and in simple presbyopia it may be all right to save patients the expense attending an examination by an oculist, but in most other conditions this should be insisted upon, for in this examination to get the best results the examiner should understand not only the science of optics but also that of medicine.

